# Longitudinal Development of Hormone Levels and Grey Matter Density in 9 and 12-Year-Old Twins

**DOI:** 10.1007/s10519-015-9708-8

**Published:** 2015-02-07

**Authors:** Rachel M. Brouwer, M. M. G. Koenis, Hugo G. Schnack, G. Caroline van Baal, Inge L. C. van Soelen, Dorret I. Boomsma, Hilleke E. Hulshoff Pol

**Affiliations:** 1 Department of Psychiatry, Brain Center Rudolf Magnus, University Medical Center Utrecht, House A.01.126, PO Box 85500, 3508 GA Utrecht, The Netherlands; 2 Department of Biological Psychology, VU University Amsterdam, Amsterdam, The Netherlands; 3 Department of Biostatistics and Research Support, Julius Center, University Medical Center Utrecht, Utrecht, The Netherlands

**Keywords:** Longitudinal, Twins, Puberty, Grey matter, Hormones, FSH, Estradiol

## Abstract

Puberty is characterized by major changes in hormone levels and structural changes in the brain. To what extent these changes are associated and to what extent genes or environmental influences drive such an association is not clear. We acquired circulating levels of luteinizing hormone, follicle stimulating hormone (FSH), estradiol and testosterone and magnetic resonance images of the brain from 190 twins at age 9 [9.2 (0.11) years; 99 females/91 males]. This protocol was repeated at age 12 [12.1 (0.26) years] in 125 of these children (59 females/66 males). Using voxel-based morphometry, we tested whether circulating hormone levels are associated with grey matter density in boys and girls in a longitudinal, genetically informative design. In girls, changes in FSH level between the age of 9 and 12 positively associated with changes in grey matter density in areas covering the left hippocampus, left (pre)frontal areas, right cerebellum, and left anterior cingulate and precuneus. This association was mainly driven by environmental factors unique to the individual (i.e. the non-shared environment). In 12-year-old girls, a higher level of circulating estradiol levels was associated with lower grey matter density in frontal and parietal areas. This association was driven by environmental factors shared among the members of a twin pair. These findings show a pattern of physical and brain development going hand in hand.

**Electronic supplementary material:**

The online version of this article (doi:10.1007/s10519-015-9708-8) contains supplementary material, which is available to authorized users.

## Introduction

Pubertal sex steroids have organizational and activating effects on brain structure, and on structural and functional connectivity (see reviews by Sisk and Zehr 2005; Peper et al. 2011a, b; Ladouceur et al. 2012). The study of healthy pubertal brain development is considered to be important in the light of the age of onset of psychiatric diseases. Several psychiatric diseases show their first symptoms during puberty and adolescence (Kessler et al. 2007) and it is thought that in this sensitive period, aberrant brain development occurs (Insel 2010). Gonodal hormones, as well as adrenal hormones, are thought to be important for teenage mental health (Marceau et al. 2014).

Several studies have found associations between pubertal hormones and brain structure (Peper et al. 2008, 2009a; Perrin et al. 2008; Neufang et al. 2009, Bramen et al. 2011, 2012; Herting et al. 2011; Nguyen et al. 2013a, b; Koolschijn et al. 2014; Herting et al. 2014). However, findings differ considerably between studies. Cross-sectionally, testosterone levels have been positively associated with whole brain size (boys; Peper et al. 2009a) and white matter volume (boys; Perrin et al. 2008; both sexes; Herting et al. 2014), amygdala (sexes combined; Neufang et al. 2009; sex × testosterone × time interaction; Herting et al. 2014) and hippocampal volume (sexes combined; Neufang et al. 2009) and have been negatively associated with cortical grey matter (girls; Bramen et al. 2011) and cortical volume (sexes combined; ACC and OFC, Koolschijn et al. 2014). Changes in testosterone levels have been associated with both thinning of the cortex (boys) and thickening of the cortex (girls) (Nguyen et al. 2013a). The few studies measuring estradiol and brain structure have found higher estradiol levels associated with smaller grey matter density and grey matter volume (girls; Peper et al. 2009a), smaller ACC volume (both boys and girls; Koolschijn et al. 2014), larger parahippocampal volume (sexes combined; Neufang et al. 2009) and smaller white matter volume and right amygdala (girls, Herting et al. 2014). Zooming in on white matter, a positive association between fractional anisotropy and testosterone has been found in boys and a negative associating between estradiol and fractional anisotropy in girls (Herting et al. 2011). These findings are not easily summarized, possibly due to the fast changes that adolescents undergo in both hormone levels and brain structure during adolescence. The strong interaction between age and pubertal development and differences in timing of puberty between the sexes may also play a role. Longitudinal studies combining pubertal development and brain maturation may provide a clearer picture. Thus far, these studies have mainly focussed on testosterone (Nguyen et al. 2013a; Herting et al. 2014; in combination with dehydroepiandrosteron Nguyen et al. 2013b), and testosterone receptors (Raznahan et al. 2010). The effect of estradiol on brain structure has been studied in a longitudinal way only recently (Herting et al. 2014): estradiol predicted white matter and right amygdala growth, and grey matter volume decrease in both boys and girls.

Here we investigate the associations of puberty-related hormones and brain structure in a longitudinal twin sample at ages 9 and 12 years. We acquired MRI brain images and assessed circulating luteinizing hormone (LH), follicle stimulating hormone (FSH), and estradiol levels in urine, and testosterone levels in saliva. Our previous work in this sample has shown that genetic factors influence (a) gray and white matter density of the brain at age 9 (Peper et al. 2009b); (b) fractional anisotropy in the main fiber tracts at age 9 and 12 (Brouwer et al. 2010b, 2012); (c) the extent to which brain volume (van Soelen et al. 2013) and cortical thickness (van Soelen et al. 2012a) changes between the age of 9 and 12 years. Variation in hormone levels were also mainly driven by genes at both ages, the exception being estradiol in girls, which seems to be influenced by a common environmental factor (Koenis et al. 2013). At age 9, a positive association between luteinizing hormone (LH) and white matter density in areas covering the splenium, left cingulum and bilateral middle temporal gyrus was seen in both sexes combined, but no correlations between the other hormones and the brain were found (Peper et al. 2008). Three years later at age 12, variation in hormone levels increased substantially, and more children had entered puberty. Here we explore the relationship between pubertal hormones and grey matter density in a genetically informative longitudinal design. We expect that higher hormone levels are associated with a more mature brain, i.e. lower cortical grey matter density. Decreasing cortical gray matter density is a process that occurs throughout childhood and adolescence. Cortical regions associated with basic functions such as vision and motor skills mature earlier than regions associated with higher order functions (Gogtay et al. 2004). However, the biological mechanisms underlying cortical thinning as observed with MRI are not clear: Cortical thinning is thought to reflect either myelination of fiber bundles close to the cortex (Paus et al. 2001), or synaptic pruning. The latter idea is based on the use-it-or-lose-it principle: dendrites and synapses that are not essential may be removed during development (Huttenlocher et al. 1982).

The twin sample allows (Boomsma et al. 2002) assessing whether associations among measures of grey matter density in the brain and hormone levels can be attributed to variations in the genome (genetic pleiotropy) or can be attributed to environmental factors that influence both phenotypes.

## Methods

### Subjects

Twins families from the Netherlands Twin Registry (van Beijsterveldt et al. 2013) were recruited for the BrainSCALE project (van Soelen et al. 2012b). At the first assessment at age 9 years (mean 9.2, SD 0.11 years), 190 twin subjects (99 females/91 males; 21 complete MZF pairs, 16 complete DZF pairs, 17 complete MZM pairs, 16 complete DZM pairs; 19 females and 14 males part of an opposite sex twin pair) underwent an extensive MRI protocol at the University Medical Center Utrecht, as was described before (Peper et al. 2009a, 2009b; van Soelen et al. 2012b). Exclusion criteria consisted of having a pacemaker, any metal material in the head and a known history of any psychiatric illness or major medical condition. Urine and saliva samples were collected on two consecutive weekdays for assessment of hormonal levels. Three years later, 125 participants returned for follow-up measurements at the University Medical Center Utrecht (mean age of twins 12.1, SD 0.24 years) (59 females/66 males; 10 complete MZF pairs, 10 complete DZF pairs, 13 complete MZM pairs, 8 complete DZM pairs, 11 females and 13 males part of an opposite sex twin pair). 113 children were scanned twice (60 boys, 53 girls). Zygosity of same-sex twins was confirmed by genome-wide SNP data. Both parents and children gave written informed consent to participate in the study. The study was approved by the Central Committee on Research involving Human Subjects of the Netherlands (CCMO) and was in agreement with the Declaration of Helsinki (Edinburgh amendments).

### Hormonal measurements

At both assessments, luteinizing hormone (LH), follicle stimulating hormone (FSH) and estradiol levels were determined in first morning urine on two consecutive days, by means of competitive immunometric luminescence assays (Architect, Abbott Laboratories, Diagnostic Division Abbott Park, Illinois, USA). Testosterone levels were obtained from saliva sampled after waking up, which were collected on the same days as the morning urine samples (competitive immunometric luminescence assay, IBL Hamburg, Germany). The lower limits for detection were 0.11 U/l (units/liter) for FSH, 0.1 U/l for LH, 150 pmol/l for estradiol and 11 pmol/l for testosterone. Hormonal levels were assessed by the endocrinological laboratory of clinical chemistry of the VU Medical Center in Amsterdam. At age 9, none of the subjects had attained menarche. At age 12, 16 girls had attained menarche of which 1 reported a regular cycle. None of the participants used oral contraceptives.

LH, FSH and total estradiol levels were divided by creatinine levels, thus correcting for variations in urine excretion rate (Kesner et al. 1998). All hormonal levels were averaged over the 2 days (if present for both days). Some children had hormone levels below detection limit on one or both days (Table 1). When data were available on 1 day only, these were entered into the analyses. The distributions of these levels were highly skewed to the left and log-transformed data were entered in all subsequent analyses. After transformation, hormonal data satisfied Mardia’s test for multivariate normal distributions (p-skewness 0.22/0.19, p-kurtosis 0.29/0.06 in girls and boys, respectively).

**Table 1:** Demographics

	First assessment Age 9		Second assessment Age 12	
	Female	Male	Female	Male
Number of twin subjects	99	91	59	66
Mean age [years, (range)]	9.2 (9.0–9.6)	9.2 (9.0–9.6)	12.1 (11.7–13.1)	12.1 (11.7–13.1)
MZ subjects (complete pairs)	43 (21)	39 (17)	26 (10)	30 (13)
DZ subjects (complete pairs)^a^	56 (16)	52 (16)	33 (10)	36 (8)
Total brain volume (ml) (SD)	1,264.1 (85.5)	1,407.5 (92.8)	1,272.1 (96.0)	1,423.5 (91.7)
Cerebral grey matter volume (ml) (SD)	673.1 (49.4)	737.1 (50.4)	655.4 (54.8)	725.7 (50.3)
Cerebral white matter volume (ml) (SD)	430.7 (39.7)	493.8 (45.0)	454.4 (45.4)	520.8 (46.7)
LH (U/mmol creatinine) (SD)^b^	0.02 (0.01)	0.02 (0.03)	0.21 (0.22)	0.18 (0.14)
Number of subjects below detection limit on 0/1/2 days	28/20/51	33/26/33	53/3/3	62/3/1
FSH (U/mmol creatinine) (SD)^b^	0.47 (0.33)	0.27 (0.18)	0.92 (0.51)	0.43 (0.33)
Number of subjects below detection limit on 0/1/2 days	98/0/0	90/1/0	59/0/0	66/0/0
Estradiol (pmol/mmol creatinine) (SD)^b^	120.8 (81.3)	135.5(124.9)	344.2 (238.1)	203.2 (136.1)
Number of subjects below detection limit on 0/1/2 days	97/2/0	87/2/0	58/1/0	66/0/0
Testosterone (pmol/l) (SD)^b^	31.1 (22.7)	26.1 (26.8)	59.6 (40.1)	68.2 (82.4)
Number of subjects below detection limit on 0/1/2 days	85/7/3	82/6/3	58/1/0	57/6/3

*MZ* monozygote, *DZ* dizygote, *LH* luteinizing hormone, *FSH* follicle stimulating hormone

^a^At age 9, 19 females/14 males were part of an opposite-sex twin pair. At age 12, 11 females/13 males were part of an opposite-sex twin pair

^b^ Means and standard deviations are given for the group of children that produced levels above the detection limits. See (Koenis et al. 2013) for more information on the hormone levels and their (genetic) associations

Tanner stages were assessed using the Tanner scales of development (Tanner 1962) through a physical exam by a trained researcher. If children were uncomfortable with the procedure, they were asked to point out their status on photographs, which were accompanied by an oral explanation of the researcher (only at age 12, 30 % boys, 15 % girls). A subset of children completed a self-report and a physical exam, and ICC values ranged between 0.73 and 0.77 except for genital development in boys (0.44). When available, we used the data as assessed by the researcher. The genetic analyses of variation in the hormone levels and associations between hormones levels and Tanner stages have been published elsewhere (Koenis et al. 2013).

### MRI acquisition and voxel based morphometry

For both assessments, structural magnetic resonance images were made on a 1.5 Tesla Philips Achieva scanner (Philips, Best, the Netherlands) using the same protocol. A three-dimensional T1-weighted scan (Spoiled Gradient Echo; TE = 4.6 ms; TR = 30 ms; flip angle 30°; 160–180 contiguous coronal slices of 1.2 mm; in-plane resolution 1 × 1 mm^2^; acquisition matrix 256 × 256) of the whole head was acquired of each subject. Intracranial masks were obtained as described in (van Soelen et al. 2013). Each voxel in the intracranium was segmented into fractions of grey matter, white matter and cerebrospinal fluid using partial volume segmentation (Brouwer et al. 2010a). This segmentation was used to obtain estimates of total brain volume, and grey and white matter volume of the cerebrum. After segmentation, images were blurred with a 3D Gaussian kernel with full-width half-max of 8 mm and subsequently warped into model space: a linear transformation to the model brain [based on optimizing a joint entropy mutual information metric (Maes et al. 1997)] was followed by nonlinear transformations with a precision up to 4 mm (ANIMAL, Collins et al. 1995). The model brain was created from the T1-weighted images of the twins and their older siblings in this study at baseline, as was described before in (Peper et al. 2008). Finally, voxels were resampled to 2 × 2 × 2.4 mm^3^ to increase statistical power. The remaining images represent the local presence, or so-called *density* of grey matter. Magnetic resonance imaging and post-processing of the MRI data was done at the University Medical Center, Utrecht.

### Statistical analysis

**Fig. 1:**
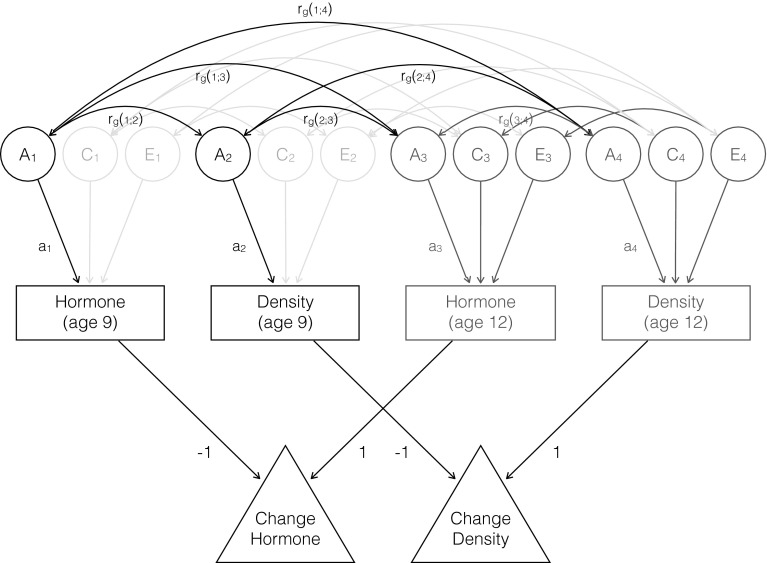
Path diagram representing the longitudinal genetic model fitted to the data collected at age 9 and 12. For simplicity, only the diagram for twin 1 is shown. The genetic components A_1_ to A_4_ of twin 1 are connected to those of twin 2 with a correlation of 1 in monozygotic twins, and 0.5 in dizygotic twins. The common environmental components C_1_ to C_4_ of twin 1 are connected to those of twin 2 with a correlation of 1 by definition. For visualization purposes, the common and unique environmental correlations and paths are not labeled. The submodel that was used to investigate the association between estradiol and grey matter density at age 12 only is *colored green* (Color figure online)

Analyses were carried out for boys and girls separately, as hormone production and subsequent physical (brain) changes may be assumed to be sex-specific. All available data were entered into the analyses, regardless whether subjects participated at age 9, age 12, or both. All analyses were implemented in OpenMx (Boker et al. 2011). Missing data were handled using the full information maximum likelihood procedure. We estimated correlations between changes in hormone level and changes in grey matter density in each voxel in a longitudinal design (Fig. 1), for each hormone and for boys and girls separately. In this model, the variance of the latent change variable V_chGM_ “change of grey matter density” is modeled as Var_(density age 9)_ + Var_(density age 12)_ – 2Cov_(density age 9,density age 12)_ = a_2_^2^ + c_2_^2^ + e_2_^2^ + a_4_^2^ + c_4_^2^ + e_4_^2^ – 2(a_2_*r_g(2,4)_*a_4_ + c_2_*r_c(2,4)_*c_4_ + e_2_*r_e(2,4)_*e_4_). Variance of latent change in hormone level V_chH_ was defined likewise. The covariance between change in grey matter density and change in hormone was modeled as Cov_(chGM,chH)_ = Cov_(hormone age 12, density age 12)_ + Cov_(hormone age 9, density age 9)_ – Cov_(hormone age 9, density age 12)_ – Cov_(hormone age 12, density age 9)_ = a_3_*r_g(3,4)_*a_4_ + c_3_*r_c(3,4)_*c_4_ + e_3_*r_e(3,4)_*e_4_ + a_1_*r_g(1,2)_*a_2_ + c_1_*r_c(1,2)_*c_2_ + e_1_*r_e(1,2)_*e_2_ – a_1_*r_g(1,4)_*a_4_ – c_1_*r_c(1,4)_*c_4_ – e_1_*r_e(1,4)_*e_4_ – a_2_*r_g(2,3)_*a_3_ – c_2_*r_c(2,3)_*c_3_ – e_2_*r_e(2,3)_*e_3_. We first tested whether change in hormone level was associated with change in grey matter density by constraining the correlation Cov_(chGM,chH)_/sqrt(V_chGM_*V_chH_) to be zero. Minus twice the difference of log-likelihoods of these models was distributed as a Chi-square distribution with one degree of freedom. Then, we tested in the same longitudinal model whether the hormone and density were associated at age 12 by constraining Cov_(hormone age 12, density age 12)_ to equal zero. Associations at age 9 have been published before (Peper et al. 2008). For each of these analyses, we corrected for multiple comparisons using the False Discovery Rate (FDR) (Genovese et al. 2002) at an FDR level of 0.05.

For the associations that reached whole brain significance (i.e. significant after FDR correction for multiple comparisons), we investigated whether the association was driven by genes or environment. Twin data allows for disentanglement of these factors, which follows from comparing monozygotic (MZ) and dizygotic (DZ) twin pairs. MZ pairs have the same genetic make-up, while DZ pairs on average share 50 % of their segregating genes. If cross-trait/cross-twin correlations (e.g. the correlation between grey matter density of twin 1 and hormone level of twin 2) are larger in MZ twins than in DZ twins, there is a genetic component to the association. If this correlation is less than twice as large in MZ twins compared to DZ twins, there is a common environmental correlation between the two traits. Finally, it is possible that a unique environmental component drives the association between two traits. In that case, there is a correlation between the two traits, but only within persons (and not between members of a twin pair. The extent to which the genetic or environmental correlations explain the phenotypic correlation between two traits, depends on the etiology of the traits, as for example, the genetic correlation is weighted by the square root of the heritabilities. We therefore also computed the rph-a, rph-c and rph-e: rph-a can be seen as the correlation that would have been observed if only genetic factors play a role and is defined as the covariance between two trait due to genetic factors, divided by the sqrt of variances of those two traits. In case of the change between hormone level and change in grey matter density, this equals (a_3_*r_g(3,4)_*a_4_ + a_1_*r_g(1,2)_*a_2_ – a_1_*r_g(1,4)_*a_4_ – a_2_*r_g(2,3)_*a_3)_/sqrt(V_chGM_*V_chH_). rph-c and rph-e are defined likewise (Toulopoulou et al. 2007).

As a post hoc analysis, we investigated whether our results could be driven by the (small) differences in age at each measurement or scanning interval, we repeated the analyses in whole brain significant voxels with a correction for age at each measurement separately.

## Results

**Fig. 2:**
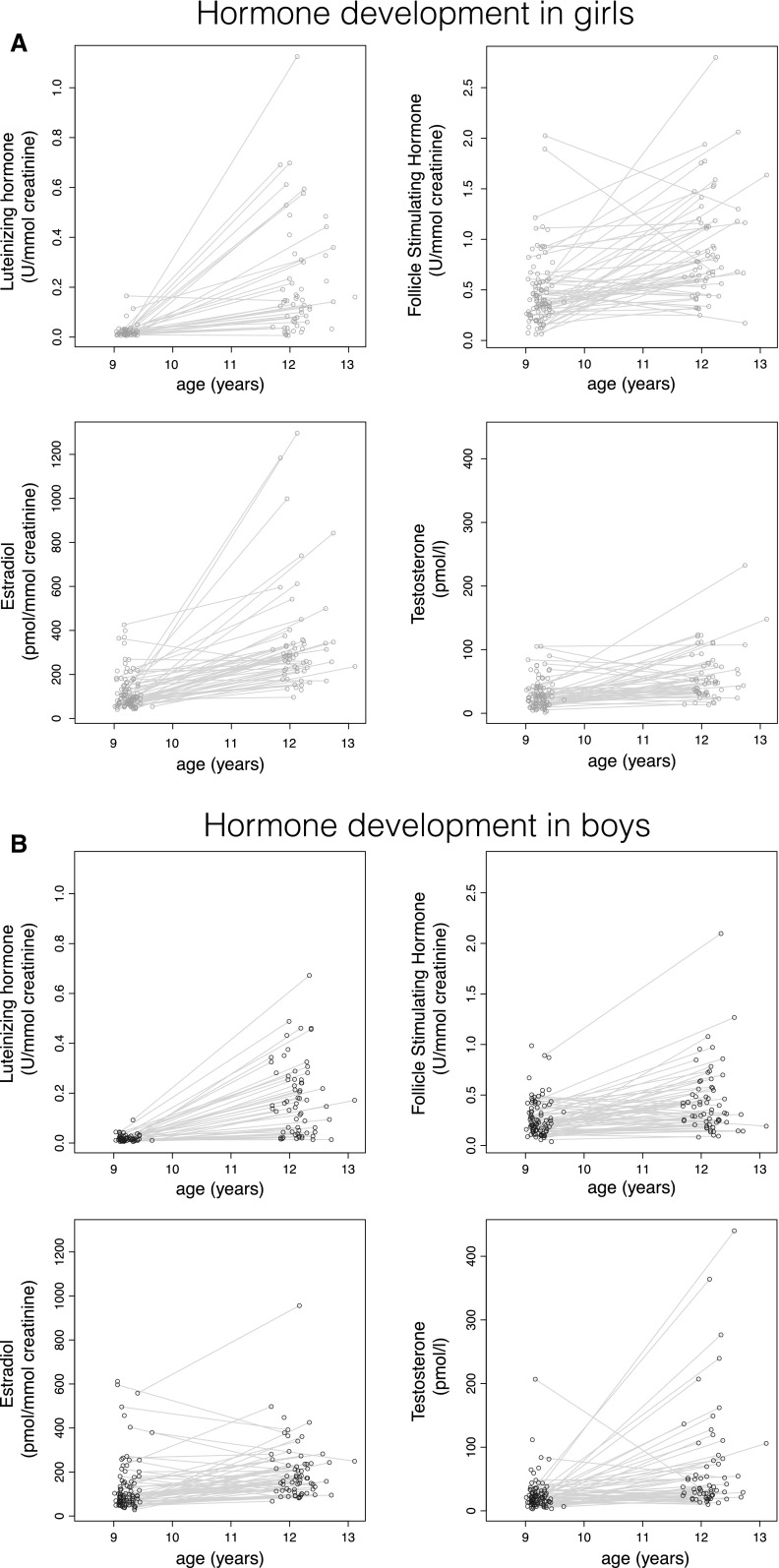
Changes in hormone levels over time in girls (**a**) and boys (**b**) (Color figure online)

Demographic characteristics can be found in Table 1. Hormone levels increased for all hormones in both boys and girls (*p* < 0.001; see Fig. 2a, b). Correlations between hormones have been described previously in (Koenis et al. 2013). In summary, in boys of age 9, LH correlated with FSH (r = 0.62). At age 12, LH correlated with all the other hormones (r ~ 0.5). In girls of age 9, LH, FSH and estradiol correlated with each other (ranging from 0.2 to 0.6). At age 12, LH correlated with all other hormones (r ~ 0.45) (Koenis et al. 2013). The distribution of Tanner stages can be found in Supplemental Figure 1. Cross-sectionally, there were no significant correlations between hormone levels and total, grey, or white matter volume at either age 9 or age 12.

### Longitudinal analysis change in hormone levels and change in grey matter density

**Fig. 3:**
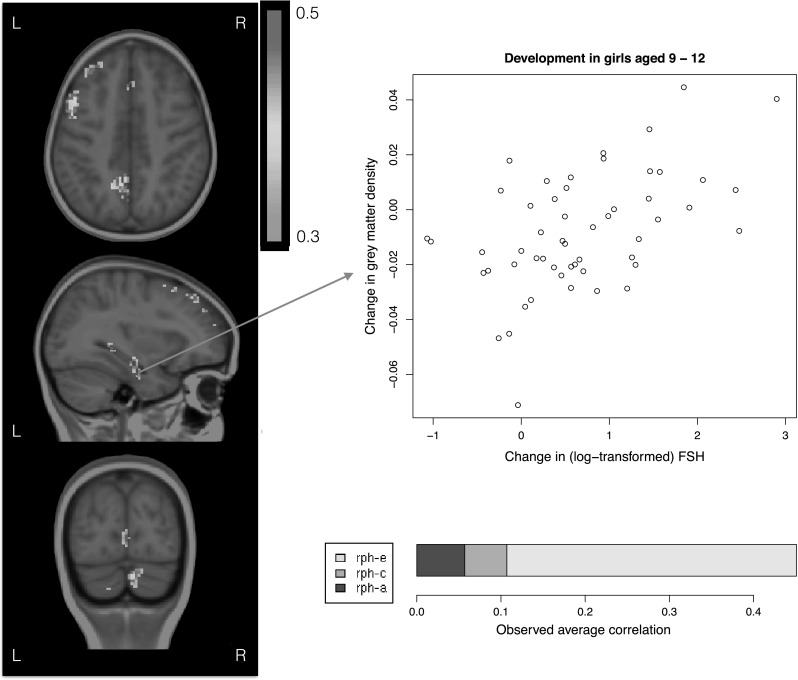
Significant positive associations between changes in *grey* matter density and changes in FSH levels in girls (FDR corrected; alpha = 0.05). Clusters of positive associations were found in the left hippocampus (*middle panel* *inset*), left frontal areas (*top and* *middle panel*), left precuneus (*top and bottom panel*), and right cerebellum (*bottom panel*). *Bottom right* the average correlation between changes in *grey* matter density and changes in FSH split up into the contribution of genetic influences shared by the two phenotypes (rph-a), common environmental influences shared by the two phenotypes (rph-c) and contribution of unique environmental influences shared by the two phenotypes (rph-e). These three add up to the observed phenotypic correlation (Color figure online)

On average, hormone levels increased from ages 9 to 12, while grey matter density decreased on average. In girls, there was a significant positive association between change in FSH levels and change in grey matter density in areas covering the left hippocampus, left (pre)frontal areas, right cerebellum, and left anterior cingulate and precuneus (Fig. 3; average correlation 0.45, Critical χ^2^ = 11.60, FDR corrected at alpha level of 0.05, *df* = 1). Within these voxels, we investigated whether there was a shared genetic, common environmental or unique environmental component explaining both changes in FSH and changes in grey matter density. In 58 % of these voxels, the association was driven by an environmental factor unique to the individual, influencing both grey matter density changes and changes in FSH (Critical χ^2^ = 4.76, FDR corrected at alpha level of 0.05, *df* = 1). In the remaining 42 % of the voxels, individual contributions of genetic or environmental sources contributing to the correlation between change in estradiol and change in grey matter density did not reach whole brain significance. When correcting for the small age differences within either measurement, conclusions did not change.

There were no whole-brain significant correlations between the change in grey matter density and the change in hormone levels for the other hormones in girls, nor were any significant associations found in boys.

### Associations between hormone levels and grey matter density at age 12

**Fig. 4:**
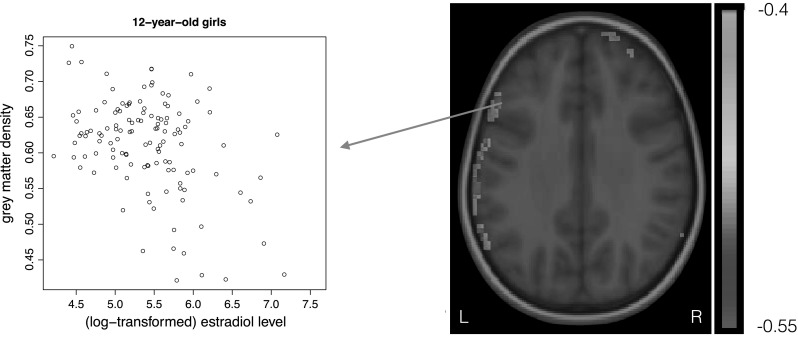
Significant negative associations between grey matter density and estradiol levels in girls at age 12 (FDR corrected; alpha = 0.05). Associations were predominantly found in (*left*) frontal and parietal areas (Color figure online)

We found significant negative correlations between grey matter density and estradiol levels in girls at age 12, in mainly left frontal and parietal cortical areas (Fig. 4; Average correlation -0.47, Critical χ^2^ = 12.58, FDR corrected at alpha level of 0.05; *df* = 1). Within these voxels, we investigated whether there was a shared genetic, common environmental or unique environmental component explaining both estradiol level and grey matter density, but these could not be disentangled, or rather, effects were not so large that they survived the correction for multiple comparisons. The nature of the results did not change when correcting for the small age differences within either measurement.

When we investigated this association in a model with fewer degrees of freedom, using a bivariate model incorporating data at age 12 only (submodel in Fig. 1), significant associations between estradiol and grey matter density at age 12 were found in the same frontal and parietal areas, but were much more widespread. A significant contribution of common environmental influences could be determined (9 % of the voxels, critical χ^2^ = 8.05, FDR corrected at alpha level of 0.05; Supplemental Figures 2 and 3). In the remaining 91 % of the voxels, individual contributions of genetic or environmental sources contributing to the correlation between estradiol and grey matter density did not reach whole brain significance.

No other associations between hormone levels and grey matter density were found at age 12 in girls. No associations were found in boys.

## Discussion

In this study we measured the influence of pubertal hormones on brain structure between 9 and 12 years of age and the extent to which these associations might be due to genetic and environmental influences. The main finding is that, in girls between the ages of 9 and 12, changes in FSH were associated with changes in areas covering the left hippocampus, left (pre)frontal areas, right cerebellum, and left anterior cingulate and precuneus. These associations were driven by unique environmental factors. Moreover, in 12-year-old girls, higher estradiol levels were associated with lower grey matter density, mainly in frontal and parietal areas. The associations between estradiol and grey matter densities were driven by common environmental influences.

Apart from our earlier work in which we showed no associations between FSH and the brain at ages 9–15, (Peper et al. 2008, 2009a), there has been little investigation of FSH in the healthy developing brain. Here, we found changes in FSH to be associated with changes in grey matter density, most interestingly in the left hippocampus. Hippocampal volumes have been shown to associate with Tanner stage in a nonlinear fashion in girls (Goddings et al. 2014) but not with sex steroid levels (Herting et al. 2014). Our results now suggest that maybe FSH is responsible for hippocampal growth in girls in the early phases of puberty. At this time, the environmental factors that explain both changes in FSH and grey matter density is unknown. There is a genetic variant for a FSH receptor gene that strongly influences the onset of puberty (Hagen et al. 2014), but our findings suggest genetic variants influencing FSH do not also influence the changes in grey matter density. Girls that are developing fast (indicating by a large increase in FSH) also obtain new (social) behavior. Indeed, early maturing girls are hypothesized to have the most adjustment difficulties because they are physically most deviant from their peers or alternatively, because they have the shortest period of time to adjust to new social and behavioral norms (Negriff and Susman 2011). Early maturation in girls has been associated with risk for adult psychopathology (Graber 2013) but also with a lower social competence (Westling et al. 2012). We may speculate that these social demands are reflected in the brain.

At age 12, we find that estradiol is negatively associated with grey matter density in girls. The associations are most prominent in the frontal and parietal brain areas. A similar pattern was seen in the older sisters of the twins in our cohort (Peper et al. 2009a). Both a higher estradiol level and less grey matter density (or cortical thinning) are signs of maturation. Here we show that the estradiol and grey matter density are related already at age 12. These findings cannot be attributed to influences of age, both of which are clearly associated with estradiol and grey matter density, since all the individuals included in the current cohort were 12 years at the time of the assessment. It is an interesting observation that much more voxels reached significance when we considered a model incorporation data from age 12 only. The longitudinal model is more flexible in the sense that there are more parameters, hence the drop in log-likelihood will be probably be smaller, leading to a smaller number of voxels that survive FDR correction for multiple comparisons.

The association between grey matter density and estradiol was mainly explained by common environmental factors shared by twins raised in the same family. As to the environmental factors that may explain the link between estradiol levels and gray matter density in girls we can only speculate. Possible candidates for this environmental source are nutrition, as body mass index has been shown to advance the start of puberty (Wagner et al. 2012), or father absence, the effect of which is moderated by ethnicity and income (Deardorff et al. 2011). Although the evidence for a shared genetic background for pubertal brain development and pubertal hormones is limited in our cohort, there are certainly genes that influence both processes. Recent studies showed effects of the number of a polymorphic trinucleotide repeat in a gene encoding for the androgen receptor in both white (Perrin et al. 2008) and grey matter (Raznahan et al. 2010). The latter study showed that a lower number of repeats, i.e. the more efficient variant, was associated with a more masculine cortical maturation pattern.

It remains an open question whether FSH or estradiol cause these changes in grey matter density. Although both FSH receptors and estradiol receptors are expressed in the brain (Hawrylycz et al. 2012), these receptors are not overly expressed in the regions in which we now observe associations between grey matter density and these hormones. Indeed, it may be that an earlier pubertal marker triggers the full cascade of both hormonal and brain changes. Examples of these include gonadotropin releasing hormone (GnRH), kiss-peptins that stimulate GnRH (Smith and Clarke 2007) or other factors triggering the growth spurt such as growth hormone, or insulin-like growth factor (Styne 2003). Another promising candidate is the steroid hormone dehydroepiandosterone (DHEA) which has been shown to associate with cortical thickness, especially in the age range between 4 and 13 years (Nguyen et al. 2013b). These authors also show an interaction effect of DHEA and testosterone on cortical thickness. It is very likely that a combination of different hormonal influences is leading to the brain changes we observe in puberty.

The idea that another hormone is implicated in both brain changes and physical maturation during puberty may also explain why thus far, no associations between estradiol and decreases of grey matter density have been found in boys. Another explanation of the absence of such an association in boys may be the delayed pubertal maturation of boys compared to girls. This delay occurs both in physical development (Mul et al. 2001) and in brain maturation (Raznahan et al. 2010) as characterized by cortical grey matter changes. The distribution of the Tanner stages (Supplemental Figure 1) shows that this is indeed the case in our cohort. The observation that the boys in our cohort are simply “too young” to show much variations in levels of hormones, specifically testosterone, may also explain the differences between our study and those of others that show associations between testosterone and the brain in boys (e.g. Raznahan et al. 2010; Nguyen et al. 2013a, b). That said, levels of sex steroids are different in boys and girls once they proceed into puberty, and this may be used as an argument that not the sex steroids, but earlier (pubertal) hormones are responsible for grey matter maturation. Another finding in favor of the view that pubertal brain changes are not driven by sex steroid production is the finding that at age 9, the children in our cohort with the first signs of secondary sexual characteristics showed decreased grey matter density in (pre)frontal and parietal areas (Peper et al. 2009b), while direct associations between the sex steroids and brain structure were not present at that time (Peper et al. 2008).

There are several limitations to take into account when interpreting the findings of our study. First, some girls (27 %) already attained menarche at age 12, but only one reported a regular cycle. It was therefore not possible to correct for variation in menstrual cycle reliably. As hormone levels and brain structure (e.g. Pletzer et al. 2010) have been shown to fluctuate with the menstrual cycle in adult women, we cannot rule out an influence of menstrual cycle on our findings. Second, the reliability of hormone level measurements decreases when the levels are closer to the detection limit (see e.g. Bay et al. 2004; Rosner et al. 2007). This may explain the lack of findings, probably more so at age 9. Third, our age range was very limited (9.0–9.6)/(11.7–13.1) at first and second assessment respectively, with 80 % of participants between (9.1–9.4) and (11.8–12.4). Nevertheless, age at scanning or differences in scanning interval may have an influence on the results. However, correlations between age at scanning/scan interval and hormone levels or brain volumes at one assessment were not significant, apart from testosterone and age in girls at the second assessment, and this correlation disappeared when removing one outlier for age, suggesting that the influence of variation in age or scanning interval is very limited in this cohort. Fourth, while being relatively large for an imaging sample in twins, the cohort is small in terms of twin-modeling. Especially the LH analyses at age 9 (with a lot of children producing below the detection limit) could have been underpowered. When interpreting the findings, one should keep in mind that absence of (genetic) associations between density and hormone levels could originate from a lack of power. Finally, while all individuals were of approximately the same age, the developmental stages may well have differed between the sexes, with boys being in an earlier developmental stage as compared to the girls (Mul et al. 2001). Thus, effects may turn up later in the boys than the girls and would therefore have been left disguised at this early age in puberty.

## Electronic supplementary material

Below is the link to the electronic supplementary material.

Schematic overview of Tanner stages at age 9 and the transition to age 12 (TIFF 3072 kb)

Significant correlations between estradiol levels and grey matter density in girls, modeling age 12 only. Associations were predominantly found in frontal and parietal areas. Because this model has much less degrees of freedom, associations are much more widespread (c.f. Figure 4) (TIFF 3072 kb)

The observed correlations (mean over significant voxels per lobe) between estradiol levels and grey matter density from the model at age 12 only split into genetic (dark grey), common environmental (grey) and unique environmental (light grey) components for each lobe. F=frontal, P=parietal, O=occipital, T=temporal, L=left, R=right. The width of the bars represent the amount of voxels that showed a significant correlation as a percentage of the number of grey matter voxels per lobe. Percentages were 21.7%; 22.4%; 5.3%; 5.9% for the left F/P/O/T lobe respectively, and 12.4%; 15.2%; 3.0% ; 2.3% for the right F/P/O/T lobe respectively. The three color bars add up to the observed correlation. F=frontal, P=parietal, O=occipital, T=temporal, L=left, R=right. (TIFF 3072 kb)
