# Supplementary figures and images for: Longitudinal Development of Hormone Levels and Grey Matter Density in 9 and 12-Year-Old Twins
Source: Behav Genet. 2015 Feb 7;45(3):313–23. doi: 10.1007/s10519-015-9708-8 (PMC4422848; doi:10.1007/s10519-015-9708-8)

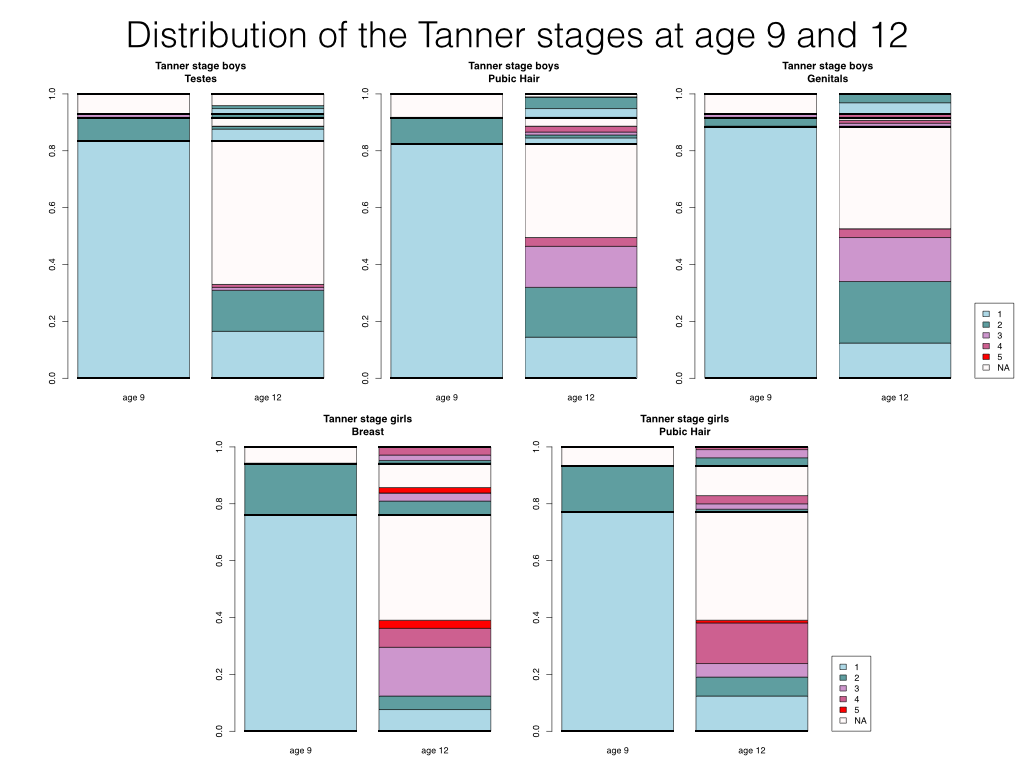

Supplement: Supplementary file 1 — Schematic overview of Tanner stages at age 9 and the transition to age 12 (TIFF 3072 kb) [file 10519_2015_9708_MOESM1_ESM.tiff]

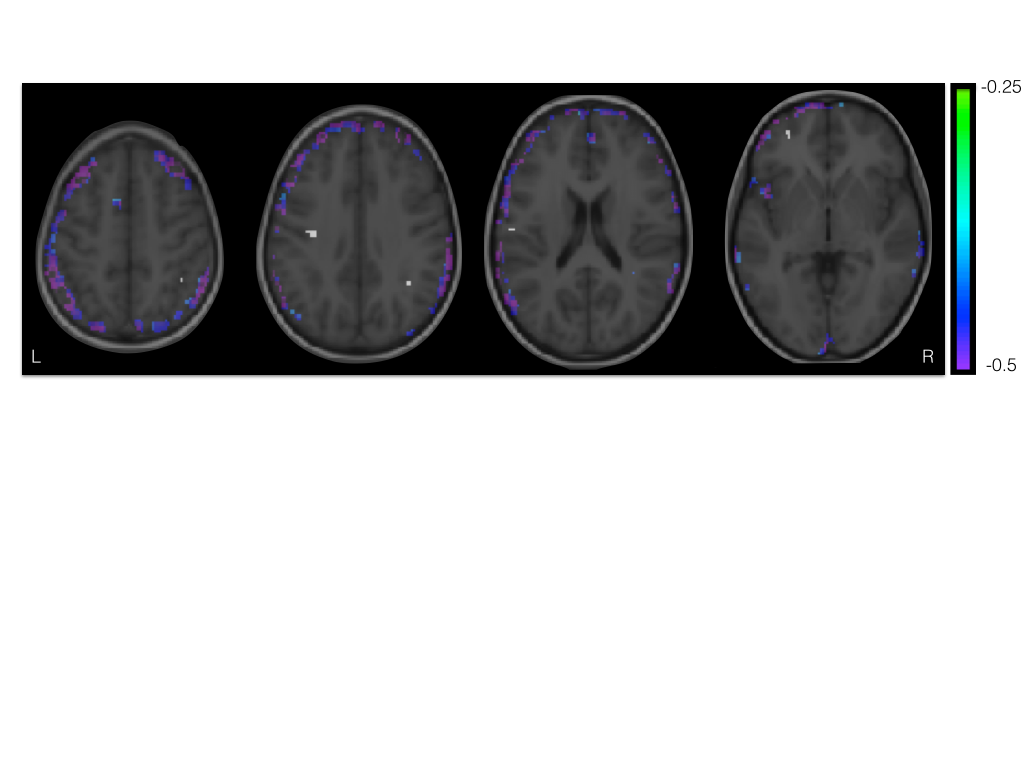

Supplement: Supplementary file 2 — Significant correlations between estradiol levels and grey matter density in girls, modeling age 12 only. Associations were predominantly found in frontal and parietal areas. Because this model has much less degrees of freedom, associations are much more widespread (c.f. Figure 4) (TIFF 3072 kb) [file 10519_2015_9708_MOESM2_ESM.tiff]

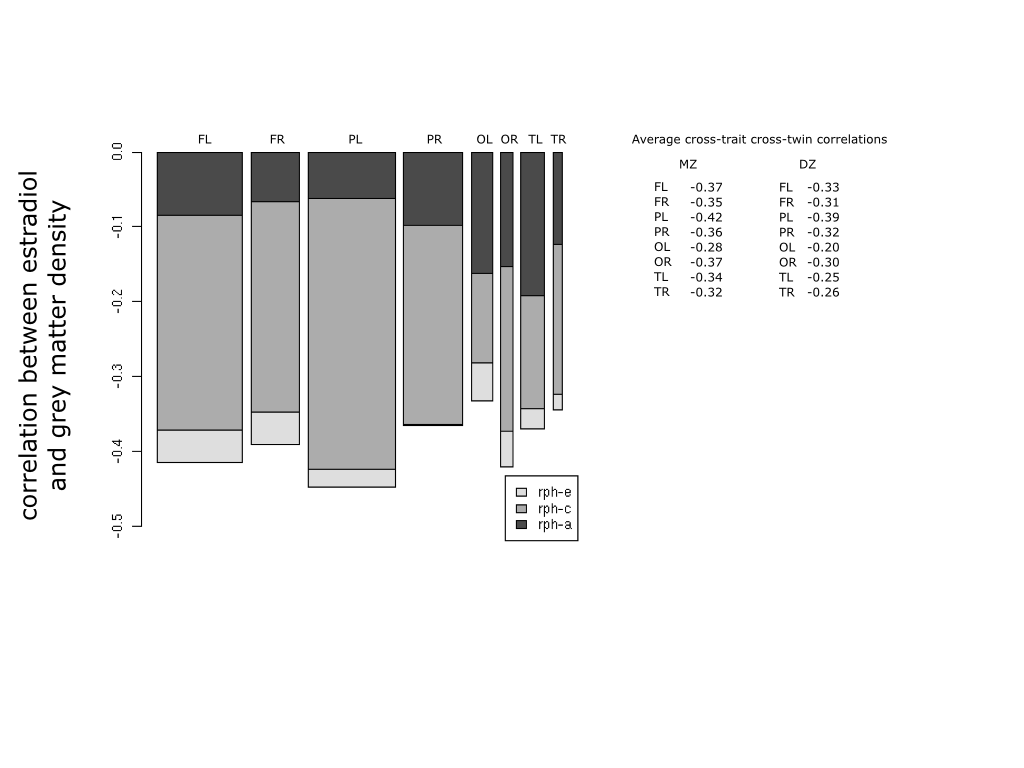

Supplement: Supplementary file 3 — The observed correlations (mean over significant voxels per lobe) between estradiol levels and grey matter density from the model at age 12 only split into genetic (dark grey), common environmental (grey) and unique environmental (light grey) components for each lobe. F=frontal, P=parietal, O=occipital, T=temporal, L=left, R=right. The width of the bars represent the amount of voxels that showed a significant correlation as a percentage of the number of grey matter voxels per lobe. Percentages were 21.7%; 22.4%; 5.3%; 5.9% for the left F/P/O/T lobe respectively, and 12.4%; 15.2%; 3.0% ; 2.3% for the right F/P/O/T lobe respectively. The three color bars add up to the observed correlation. F=frontal, P=parietal, O=occipital, T=temporal, L=left, R=right. (TIFF 3072 kb) [file 10519_2015_9708_MOESM3_ESM.tiff]
